# Engaging Community-Based Organizations in the Scale and Spread of WalkOn!

**DOI:** 10.1093/geroni/igaf122.1772

**Published:** 2025-12-31

**Authors:** Jaime Hughes, Laura Jakiela, Barbara Nicklas, Elizabeth Kemp, Courtney Hayes, Justin Moore, Case Peters, Michelle Simpson

**Affiliations:** Wake Forest University School of Medicine, Winston-Salem, North Carolina, United States; Wake Forest University School of Medicine, Winston-Salem, North Carolina, United States; Wake Forest University School of Medicine, Winston-Salem, North Carolina, United States; Atrium Health Wake Forest Baptist Medical Center, Winston Salem, North Carolina, United States; Wake Forest University School of Medicine, Winston-Salem, North Carolina, United States; Wake Forest University School of Medicine, Winston-Salem, North Carolina, United States; Wake Forest University School of Medicine, Winston-Salem, North Carolina, United States; Advocate Aurora Health, Milwaukee, Wisconsin, United States

## Abstract

Few community-based walking programs exist within rural areas. Scaling WalkOn!, an evidence-informed walking program for older adults with mobility limitations, may require adaptations to intervention components and implementation strategies to best align with the needs, preferences, and capacity of rural communities. Informed by principles of implementation science and guided by the Consolidated Framework for Implementation Research, we engaged diverse community-based organizations (CBOs) in exploring implementation determinants, organizational readiness, and program adaptations. Project invitations were sent to 93 CBOs across four diverse service sectors: faith-based, fitness/recreation, community services (e.g., libraries), and aging organizations. Twenty-four (26%) CBOs indicated interest, 13 (14%) attended an informational session, and 8 (9%) enrolled. While 7 (88%) have implemented new programs, one-quarter reported no exercise or older adult (OA) programs. Information sessions revealed CBOs’ heavy interest in adopting WalkOn! despite varied staffing models ranging from a part-time director to 250+ staff/leaders/volunteers (S/L). CBOs participated in quantitative surveys (S/L, N = 21; OA, N = 102) to assess site readiness, barriers and facilitators to adoption and sustainment, and desired program adaptations. CBOs’ motivation for adopting WalkOn! included enhancing access to evidence-based programs (N = 19, 90%) and responding to community needs (N = 15, 71%). S/L respondents reported high organizational readiness and only endorsed an average of 1.2 barriers (competing demands, funding) when asked to rank 8 potential implementation barriers. OAs’ most desired intervention adaptations included home exercise instruction (N = 53, 52%) and accountability buddies (N = 38, 37%). This session will conclude with best practices for engaging diverse CBOs in the planning, adaptation, and scale-up of effective programs.

